# Bacteriophage efficacy in controlling swine enteric colibacillosis pathogens: An *in vitro* study

**DOI:** 10.14202/vetworld.2022.2822-2829

**Published:** 2022-12-12

**Authors:** Techaphon Songphasuk, Napakhwan Imklin, Pattaraporn Sriprasong, Yonlayong Woonwong, Rujikan Nasanit, Somchai Sajapitak

**Affiliations:** 1 Veterinary Clinical Study Program, Faculty of Veterinary Medicine, Kasetsart University, Kamphaeng Saen, Nakhon Pathom 73140, Thailand; 2 Department of Biotechnology, Faculty of Engineering and Industrial Technology, Silpakorn University, Nakhon Pathom 73000, Thailand; 3 Department of Farm Resources and Production Medicine, Faculty of Veterinary Medicine, Kasetsart University, Kamphaeng Saen, Nakhon Pathom 73140, Thailand; 4 Department of Large Animal and Wildlife Clinical Sciences, Faculty of Veterinary Medicine, Kasetsart University, Kamphaeng Saen, Nakhon Pathom 73140, Thailand

**Keywords:** bacteriophages, colibacillosis, diarrhea, *Escherichia coli*, swine

## Abstract

**Background and Aim:**

Swine enteric colibacillosis caused by *Escherichia coli* is a major problem in the swine industry, causing diarrhea among swine and resulting in substantial financial losses. However, efforts to counter this disease are impeded by the increase in antimicrobial resistance (AMR) worldwide, so intensive research is being conducted to identify alternative treatments. This study isolated, characterized, and evaluated the efficacy of bacteriophages to control pathogens causative of swine enteric colibacillosis.

**Materials and Methods:**

Five sewage samples were collected from different areas of a swine farm in Suphanburi province, Thailand and the bacteriophages were enriched and isolated, followed by purification by the agar overlay method using *E. coli* RENR as the host strain. The selected phages were characterized by evaluating their morphology, while their specificity was verified by the host range test. The efficiency of plating and multiplicity of infection (MOI) were also determined.

**Results:**

Four selected phages, namely, vB_Eco-RPNE4i3, vB_Eco-RPNE6i4, vB_Eco-RPNE7i1, and vB_Eco-RPNE8i3, demonstrated different patterns of host range and phage efficiency. They significantly decreased *E. coli* concentration at the tested MOIs (0.01–100) from 1 h onward. However, bacterial regrowth was observed in all phage treatments.

**Conclusion:**

This study shows the potential of using phages as an alternative treatment for swine enteric colibacillosis. The obtained results demonstrated that the selected phages had a therapeutic effect against pathogens causative of swine enteric colibacillosis. Therefore, phages could be applied as an alternative treatment to control specific bacterial strains and reduce AMR arising from the overuse of antibiotics.

## Introduction

Swine enteric colibacillosis is a common disease of suckling and weaning pigs caused by infection with *Escherichia coli* in the small intestine. *Escherichia coli*, a Gram-negative bacterium, is a well-known causative agent of neonatal and post-weaning diarrhea, particularly enterotoxigenic *E. coli* (ETEC). Enterotoxigenic *E. coli* is the most common pathotype, which produces one or more enterotoxins that induce secretory diarrhea. It has a major economic impact on the swine industry, with losses due to acute death, weight loss, and the high cost of treatments, vaccinations, and feed supplements [1]. *Escherichia coli* bacteria are widespread in both pig fecal microbiota and the farm environment [2, 3]. Current preventive disease strategies depend on biosecurity management and treatment guidelines recommending antibiotics and dietary supplements such as prebiotics and probiotics [4, 5]. Antibiotics are currently the first choice for treating bacterial infections, with various prophylactic and metaphylactic applications commonly used in many countries [6]. However, this has led to dramatic increases in antimicrobial resistance and greater transfer of resistance genes worldwide [7, 8]. Against this background, increasing attention has been drawn to alternative treatments, especially the use of bacteriophages as bacterial viruses to eliminate specific bacteria. Bacteriophages (or simply “phages”) are abundant in a variety of environments, including food products, wastewater, soil, and forest undergrowth [9]. Phages are ubiquitously detected alongside bacteria and require bacterial cells as hosts to sustain life [10]. Specific bacteria that serve as hosts for phage replication are damaged by the mechanisms of phage–host interaction. These mechanisms affect both biological and physical factors of the bacterial host [11, 12]. Phage therapy is now recognized as an alternative method for treating bacterial infections, which can limit the need to use antibiotics.

Some researchers have questioned the value of using phages to counter pathogenic bacteria. For example, Wongsuntornpoj *et al*. [13] reported the phage–host specificity between Thai and U.S. phages. The results revealed that phages isolated from Thailand had broader host ranges than those isolated from the U.S. The abundance of the host is one of the most important factors determining the phage–host range. Phages isolated from environments in which their host is present at a high density typically have narrow host ranges or are specialized for conditions of host abundance [14]. Moreover, the different characteristics of phages have been shown to be associated with various veterinary and human healthcare regimes in different continents.

Phage–host specificity is one of the major factors limiting the potential value of phage therapy. Therefore, this study aimed to isolate, characterize, and evaluate the efficacy of *E. coli*-specific bacteriophages to control pathogens causative of swine enteric colibacillosis.

## Materials and Methods

### Ethical approval

The study was approved by the Ethical Committee, Kasetsart University, Thailand (ACKU65-VET-064). Kamphaeng Saen Veterinary Diagnostic Center provided *E. coli* strains of pig.

### Study period and location

The study period was conducted from August 2020 to December 2021 at the Faculty of Veterinary Medicine, Kasetsart University, Thailand, and Department of Biotechnology, Silpakorn University, Thailand.

### Bacteria and culture conditions

Two groups of *E. coli* were used in this study. The first group consisting of 13 *E. coli* RENR strains, was employed for phage isolation, initial screening, host range testing, and efficiency of plating (EOP) assay, while the second group consisted of 14 *E. coli* M strains . These two groups of strains were obtained from Kamphaeng Saen Veterinary Diagnostic Center, Other four bacterial strains, namely, *Klebsiella pneumoniae* klpnks 648, *Enterobacter* spp. Enspks 513 [15], *Enterococcus faecalis* SR14, and *E. faecalis* AIM06 [16] were used for host range testing and EOP assay. All bacterial strains were cultured in tryptic soy broth (TSB) at 37°C for 16–18 h before the experiment.

### Isolation, purification, and screening of phages

Phage isolation and purification were conducted as described by Imklin and Nasanit [17]. Briefly, five sewage samples were collected from a swine farm in Suphan Buri Province, Thailand. The large particles were first removed by centrifugation and the samples were then mixed with 10×TSB and bacterial hosts. The mixtures were incubated at 37°C overnight, followed by centrifugation to obtain the phage supernatant. Subsequently, the presence of phages in the supernatant was confirmed by the agar overlay assay with each host strains. After incubation overnight, the observed plaques were picked up and soaked in SM buffer. Finally, these plaques were used as phage samples for further experiments.

Purification of isolated phages was carried out by the agar overlay method. Each phage sample (100 μL) was mixed with its specific host strain (100 μL) and 3.5 mL of top agar (0.45% agar). The mixture was then poured onto a tryptic soy agar (TSA) (HiMedia, India) plate and incubated overnight. A single plaque was picked up and the purification steps were repeated at least 3 times. The purified phages were initially observed using the spot test technique. Briefly, the bacterial host was agar overlaid on a TSA plate, then 20 μL of the phage suspension were dropped and incubated overnight at 37°C.

To further screen and characterize the phages, each bacterial lawn was prepared by pouring a mixture of bacterial culture and molten agar on a TSA plate. Each purified phage sample was spotted on bacterial lawns and incubated overnight at 37°C. The appearances were documented regarding the transparency of zones and the count ability of plaques (+++, confluent lysis; ++, semi-confluent lysis; and +, individual plaques). The ETEC-specific phages and phages with broad infectivity were randomly selected for further experiments.

### Host range determination and EOP assay

Host range and EOP tests against 31 bacterial strains were performed. For the host range test, each selected phage was mixed with each bacterial strain and the mixture was subjected to agar overlay assay. The plates were incubated at 37°C for 16–18 h. The results were recorded as positive (+) when plaques appeared on plates and negative (–) when they did not.

For the EOP assay, the selected phages were serially diluted with SM buffer before performing the same procedure as used for the host range test. After incubation, the plaques were counted and the EOP values were calculated by dividing the phage titer on the tested strain by the phage titer on the host strain. The values were defined as follows: <0.001, inefficiency; 0.001–0.2, low efficiency; 0.2–0.5, medium efficiency; and >0.5, high efficiency.

### Bacterial challenge tests by the multiplicity of infection (MOI)

The multiplicity of infection is a feature of phages that usually determines the effective ratio of phages to bacterial host cells for bacterial reduction. Phages with effective EOP values were selected for the MOI assay. This assay was conducted by mixing each phage suspension in TSB with mid-log-phase *E. coli* (10^8^ CFU/mL) to reach MOI values of 0.01, 1, and 100 in a total volume of 30 mL. The mixtures were incubated with shaking (37°C, 125 rpm) for 6 h. One milliliter of each sample was collected to examine the bacterial and phage titers every hour. Briefly, each sample was then separated into two fractions. The first fraction was spotted on TSA plates and incubated at 37°C overnight to enumerate the bacterial colonies, while the second fraction was diluted with SM buffer. Then, the agar overlay method was performed to enumerate the plaques. The bacterial and phage titers were calculated as CFU/mL and PFU/mL, respectively.

### Characterization of phage morphology

Phage stock with a high titer (~10^9^ PFU/mL) was used to evaluate the morphology using a Hitachi Hightech HT7700 Transmission Electron Microscope (Hitachi, Japan). For preparation steps, a drop of phage suspension was applied onto a carbon-coated copper grid for 10 min followed by negative staining with 2% uranyl acetate for 2 min. Finally, the morphology of each selected phage was observed under a microscope at 80 kV.

### Statistical analysis

All experiments were performed in triplicate, with results reported as mean and/or standard deviation. The mixed model for repeated measures was conducted to compare the bacterial count (log CFU/mL) between the control and treatment groups at different time points, followed by Tukey’s *post hoc* analysis. All statistical analyses were carried out using GraphPad Prism 8 software (www.graphpad.com) p < 0.05 was considered statistically significant.

## Results

Thirty-two phages were isolated from five sewage samples using 13 *E. coli* host strains. Six isolated phages were specific to only their hosts, while the other 26 were effective against two to five bacterial strains (Table-1). According to the ETEC and ability to infect, five phages were randomly selected for host range and EOP assays: vB_Eco-RPNE4i3, vB_Eco-RPNE6i4, vB_Eco-RPNE7i1, vB_Eco-RPNE8i3, and vB_Eco-RPNE11i4.

**Table-1:** Host range of isolated bacteriophages by spot test.

Phage isolate	*E. coli* host strain												

	RENR1	RENR2	RENR4	RENR5	RENR6	RENR7	RENR8	RENR9	RENR10	RENR11	RENR12	RENR13	RENR14
vB_Eco-RPNE4i3	–	–	+++	+++	+++	–	–	–	–	–	–	–	–
vB_Eco-RPNE4i4	–	–	+++	+++	+++	–	–	–	–	–	–	–	–
vB_Eco-RPNE4i5	–	–	+++	+++	+++	–	–	–	–	–	–	–	–
vB_Eco-RPNE5i3	–	–	+++	+++	+++	–	–	–	–	–	–	–	–
vB_Eco-RPNE5i4	–	–	+++	+++	+++	–	–	–	–	–	–	–	–
vB_Eco-RPNE5i5	–	–	++	++	++	–	–	–	–	–	–	–	–
vB_Eco-RPNE6i3	–	–	+++	+++	+++	–	–	–	–	–	–	–	–
vB_Eco-RPNE6i4	–	–	+++	+++	+++	–	–	–	–	–	–	–	–
vB_Eco-RPNE6i5	–	–	++	++	++	–	–	–	–	–	–	–	–
vB_Eco-RPNE7i1	–	–	–	–	–	+++	–	–	–	–	–	–	–
vB_Eco-RPNE7i2	–	–	–	–	–	+++	–	–	–	–	–	–	–
vB_Eco-RPNE7i3	–	–	–	–	–	+++	–	–	–	–	–	–	–
vB_Eco-RPNE7i4	–	–	–	–	–	+++	–	–	–	–	–	–	–
vB_Eco-RPNE7i5	–	–	–	–	–	+++	–	–	–	–	–	–	–
vB_Eco-RPNE8i1	–	–	–	–	–	–	+	+	+	–	–	–	–
vB_Eco-RPNE8i2	–	–	–	–	–	–	+	+	+	–	–	–	–
vB_Eco-RPNE8i3	+	+	–	–	–	–	+++	+++	+++	–	–	–	–
vB_Eco-RPNE9i1	–	–	–	–	–	–	+	+	+	–	–	–	–
vB_Eco-RPNE9i2	–	–	–	–	–	–	+	+	+	–	–	–	–
vB_Eco-RPNE9i3	–	+	–	–	–	–	+++	+++	+++	–	–	–	–
vB_Eco-RPNE9i5	–	–	–	–	–	–	+	+	+	–	–	–	–
vB_Eco-RPNE10i1	–	–	–	–	–	–	–	–	+++	–	–	–	–
vB_Eco-RPNE10i2h	–	–	–	–	–	–	–	–	+++	+++	–	–	–
vB_Eco-RPNE10i2	–	–	–	–	–	–	–	–	++	++	–	–	–
vB_Eco-RPNE10i3	–	–	–	–	–	–	–	–	+	+	–	–	–
vB_Eco-RPNE10i4	–	–	–	–	–	–	–	–	++	+	–	–	–
vB_Eco-RPNE10i5	–	–	–	–	–	–	–	–	++	+	–	–	–
vB_Eco-RPNE11i1	+	+	–	–	–	–	–	–	++	+++	–	–	+
vB_Eco-RPNE11i2	–	–	–	–	–	–	–	–	++	++	–	–	–
vB_Eco-RPNE11i3	–	–	–	–	–	–	–	–	+	–	–	+	+
vB_Eco-RPNE11i4	–	–	–	–	–	–	–	–	+	+	–	+	+
vB_Eco-RPNE11i5	–	–	–	–	–	–	–	–	+	+	–	+	+

+++=Confluent lysis, ++=Semi-confluent lysis, +=Individual plaques, –=No lysis, *E. coli*=*Escherichia coli*

The host range results demonstrated that three phages were able to lyse two tested bacterial strains, including their hosts: vB_Eco-RPNE6i4, vB_Eco-RPNE7i1, and vB_Eco-RPNE11i4. Meanwhile, vB_Eco-RPNE4i3 was able to infect three *E. coli* strains, namely, RENR4, RENR5, and RENR6 (Table-2). Interestingly, vB_Eco-RPNE8i3 demonstrated the ability to infect five *E. coli* strains, namely, RENR8, RENR9, M158, M179, and M243. However, none of the other tested bacterial species was eradicated by these phages. For the EOP assay, the selected phages were largely highly effective against their specific bacterial strains, except for vB_Eco-RPNE11i4. Therefore, four phages were selected for further experiments: vB_Eco-RPNE4i3, vB_Eco-RPNE6i4, vB_Eco-RPNE7i1, and vB_Eco-RPNE8i3.

**Table-2:** The efficiency of plating of phages against tested bacterial strains.

Tested bacterial strain	Bacteriophage				

	vB_Eco-RPNE4i3	vB_Eco-RPNE6i4	vB_Eco-RPNE7i1	vB_Eco-RPNE8i3	vB_Eco-RPNE11i4
*E. coli* RENR1					
*E. coli* RENR2					
*E. coli* RENR4	Host				
*E. coli* RENR5	0.14	0.85			
*E. coli* RENR6	0.14	Host			
*E. coli* RENR7			Host		
*E. coli* RENR8				Host	
*E. coli* RENR9				1.30	
*E. coli* RENR10					0.067
*E. coli* RENR11					Host
*E. coli* RENR12					
*E. coli* RENR13					
*E. coli* RENR14					
*E. coli* M158				4.95	
*E. coli* M170					
*E. coli* M171					
*E. coli* M179				1.10	
*E. coli* M181			1.43		
*E. coli* M184					
*E. coli* M187					
*E. coli* M209					
*E. coli* M226					
*E. coli* M240					
*E. coli* M241					
*E. coli* M242					
*E. coli* M243				1.95	
*E. coli* M245					
*K. pneumoniae* klpnks 648					
*Enterobacter* spp. Enspks 513					
*E. faecalis* SR14					
*E. faecalis* AIM06					

EOP values: <0.001, inefficiency; 0.001−0.2, low efficiency; 0.2−0.5, medium efficiency; >0.5, high efficiency. *E. coli*=*Escherichia coli*, *K. pneumoniae*=*Klebsiella pneumoniae*, *E. faecalis*=*Enterococcus faecalis*

**Figure-1:**
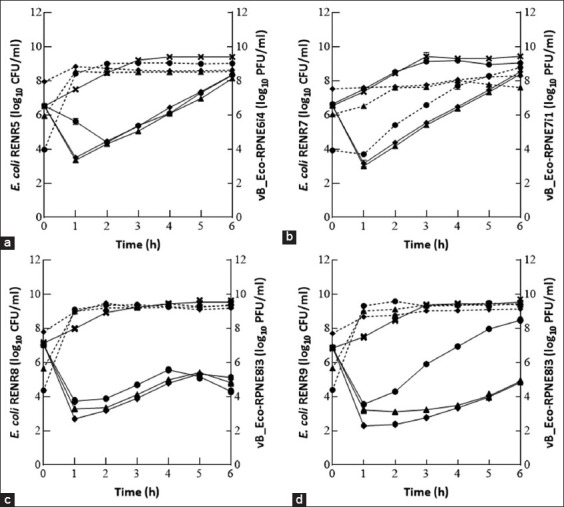
Efficacy of bacteriophage against *Escherichia coli* RENR strains in different MOI ratio. (a) vB_Eco-RPNE6i4 versus RENR5, (b) vB_Eco-RPNE7i1 versus RENR7, (c) vB_Eco-RPNE8i3 versus RENR8 and (d) vB_Eco-RPNE8i3 versus RENR9. The solid lines and dash lines represent the result of bacterial concentration and phage titer, respectively. The line symbols indicated the experimental results of bacterial control (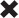), MOI 0.01 (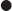), MOI 1 (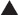), and MOI 100 (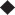). The error bar represented the standard deviation value of repeated experiments.

According to the results of MOI assay, the selected phages were examined for their ability to control specific *E. coli* strains at the MOI range of 0.01–100 (Figure-1). Phages vB_Eco-RPNE6i4 and vB_Eco-RPNE8i3 significantly reduced (p < 0.05) the growth of their specific *E. coli* strains at all tested MOIs (Figures-1a, c, and d), while vB_Eco-RPNE7i1 considerably decreased (p < 0.05) its host at MOI 1 and 100. However, in all experiments with the selected phages, bacterial regrowth was found to occur. Phage titers in most experiments increased dramatically during the 1^st^ h and remained steady from 2 to 6 h. In contrast, the phage titer continuously increased until the end of the experimental period when using vB_Eco-RPNE7i1 at MOI 0.01 (Figure-1b).

**Figure-2:**
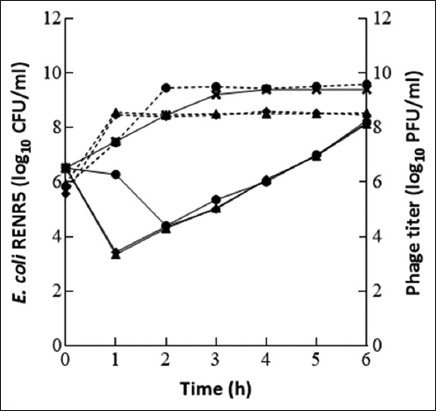
Efficacy of bacteriophages against *Escherichia coli* RENR5 at MOI 1. The result of individual phage (vB_Eco-RPNE4i3 or vB_Eco-RPNE6i4) compared with phage cocktail. The solid lines and dash lines represent the result of bacterial concentration and phage titer, respectively. The symbols indicate the experimental results of bacterial control (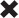), vB_Eco-RPNE4i3 (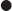), vB_Eco-RPNE6i4 (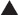), and phage cocktail (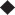). The error bar represented the standard deviation value of repeated experiments.

Two phages, vB_Eco-RPNE6i4 and vB_Eco-RPNE4i3, and their combination, were also used to test the effectiveness of individual phages and as a phage cocktail against *E. coli* RENR5 (Figure-2). These results illustrated that the phage cocktail and vB_Eco-RPNE6i4 diminished *E. coli* RENR5 by more than 2 log CFU/mL after 1 h of incubation at MOI 1. Conversely, *E. coli* RENR5 slowly declined during the first 2 h when encountering vB_Eco-RPNE4i3. After 2 h, an increase in bacterial concentration was observed in all experiments. Notably, there were no significant differences between the lytic activities of individual phages and the phage cocktail.

**Figure-3:**
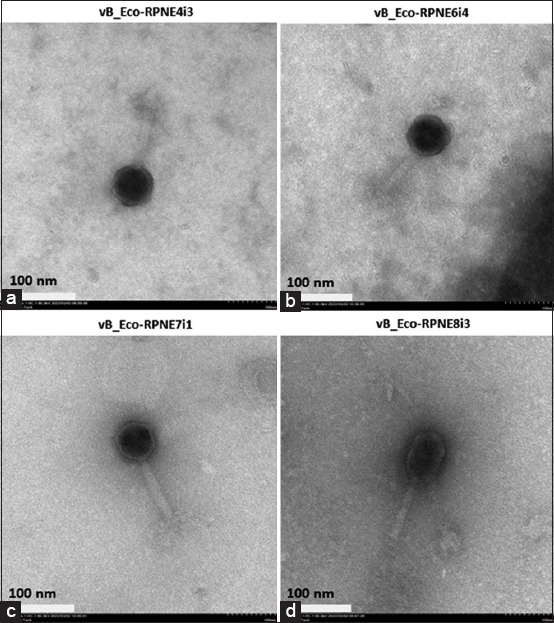
Transmission electron micrographs of *Escherichia coli* phages: (a) vB_Eco-RPNE4i3; (b) vB_Eco-RPNE6i4; (c) vB_Eco-RPNE7i1; and (d) vB_Eco-RPNE8i3.

Transmission electron microscopy (TEM) analysis illustrated that each selected phage had an icosahedral head and long tail (Figure-3). Moreover, a contractile sheath and tail fibers characteristic of myoviruses were also observed. However, there were differences in the sizes and shapes of the heads and tails, as shown in Table-3. The vB_Eco-RPNE4i3 phage possessed a wider head and a longer tail than the others, while the vB_Eco-RPNE8i3 phage had the longest head.

**Table-3:** Morphology of the four *E. coli* phages isolated in this study.

Morphology	Bacteriophage			

	vB_Eco-RPNE4i3	vB_Eco-RPNE6i4	vB_Eco-RPNE7i1	vB_Eco-RPNE8i3
Head width (nm)	75.72 ± 7.68	74.84 ± 9.26	71.20 ± 4.92	72.12 ± 6.06
Head length (nm)	81.95 ± 2.54	81.11 ± 5.50	80.43 ± 0.93	95.24 ± 3.20
Tail width (nm)	20.68 ± 2.26	17.28 ± 2.35	17.32 ± 1.74	16.44 ± 1.44
Tail length (nm)	118.64 ± 6.65	96.92 ± 5.53	112.44 ± 7.40	103.82 ± 4.02

The average size of the head and tail of each phage was calculated by measuring ten particles. *E. coli*=*Escherichia coli*

## Discussion

This study determined the efficacy of bacteriophages in controlling pathogens causative of swine enteric colibacillosis. Pathogenic *E. coli* as an etiological agent behind colibacillosis is abundant in the environment, particularly in wastewater. Phages can be easily isolated from environments in which their hosts are present. Phages specific to pathogenic *E. coli* are also found on pig farms and associated with outbreaks of colibacillosis. Kumar *et al*. [18] reported that phages isolated from poultry and pig farms could be used in the biocontrol of specific poultry and pig pathogens, while another study demonstrated that 17 specific *E. coli* phages isolated from pig farms were active against both related and unrelated ESBL/AmpC *E. coli* isolates. Interestingly, 14/17 phages were more effective against ESBL/AmpC *E. coli* isolated from turkey farms than that from pig farms [19]. This indicated that not the site of isolation but rather phage–host interaction determines the infectibility of phages.

The spot technique is the simple and most extensively used technique for determining phage–host specificity. Lysis zones on bacterial lawns show evidence of phage–host interaction. Some phages can infect only a few bacterial strains, whereas others can attack numerous species or multiple bacterial genera [20, 21]. In this study, most selected phages were capable of lysing more bacterial strains in the host range test than in the EOP assay. Moreover, some phages showed different lytic profiles when using different analytical techniques. The spot and agar overlay techniques were performed in the host range test and the EOP assay, respectively. A previous study also revealed that the average number of susceptible bacterial strains obtained from the EOP assay accounted for half of those from the spot test results [22]. The discovery of broad-spectrum phages might assist in the selection of phage candidates for a specific purpose. However, it is not sufficiently reliable to use only the host range test to evaluate the effectiveness of phages by the spot test, which is usually used for initial screening [17, 23]. Lysis zones might occur due to incomplete infection or lysis from without [24] and EOP should also be conducted to confirm phage specificity.

For phage therapy, an appropriate ratio of phages to bacteria, or MOI, is required to effectively reduce the number of bacterial cells [25, 26]. In this study, MOI of 100 was a suitable ratio of vB_Eco-RPNE8i3 to lyse bacterial cells. Higher MOI values were shown to be more effective than lower ones. A similar trend was also observed between MOI 100 and MOI 1 for some phages (Figure-1). Our findings matched the results of Nikapitiya *et al*. [27], who found that the population of *Aeromonas* *salmonicida* subsp. *salmonicida* was rapidly eliminated by ASP-1 phage at MOI 10 within 1 h, while at lower MOIs, including 1, 0.1, and 0.01 bacterial concentration decreased slightly from 2 h. The pattern of bacterial reduction remained stable from 4 h onward at all tested MOIs. Use of the appropriate dose of phages may enhance product safety, but the minimum dose is preferable to avoid the persistence of the phages in the body [28]. The minimum phage dose that is sufficient to control bacteria at non-infectious levels should be administered.

In this study, bacterial regrowth was observed in both individual phage and phage cocktail treatments (Figures-1 and 2) caused by the development of phage-resistant bacteria. Yordpratum *et al*. [29] also observed bacterial regrowth at MOI 0.1 in phage treatments, which occurred either due to bacterial host resistance to phage infection or as a result of bacterial cell debris interfering with the phage.

Phage cocktails are often used in phage therapy because combining phages promotes treatment efficacy. In this study, we investigated the effect of combining the two phages, vB_Eco-RPNE6i4 and vB_Eco_RPNE4i3, against an *E. coli* strain (Figure-2). The phage cocktail did not significantly reduce the bacterial concentration compared with the individual phages at the same MOI. Considering the EOP results, vB_Eco_RPNE4i3 had low efficiency against *E. coli* RENR5. This implied that the high efficacy of the phage cocktail was dominated by the vB_Eco-RPNE6i4. This finding matched the results of Niu *et al*. [30], who found that two phage cocktails, T5 + T4 + rV5 and T5 + rV5, were less effective than treatment with the T5 phage alone, suggesting that the combination of phages in a cocktail might influence efficacy due to antagonistic effects between phages [30]. However, Naghizadeh *et al*. [31] reported that phage cocktails reduced bacterial cells more than the T3 phage alone, indicating phage synergism. The concept of different polysaccharide depolymerized enzymes associated with phage penetration might be related to the mechanism behind phage synergism [32]. In general, phages have high specificity to their hosts, resulting in a low spectrum of action [33], which is similar to the narrow host range characteristics of the two isolated phages. Even though they were combined as a phage cocktail, their ability to achieve lysis was not improved when tested with *E. coli* RENR5. Therefore, the effectiveness of each phage should be taken into account during the preparation of phage cocktails to promote the therapeutic use of phages.

Transmission electron microscopy is a common tool for investigating phage morphology, but is insufficient for the taxonomic classification of phages. Next-generation sequencing has become a powerful tool for taxonomic classification and is commonly used to classify phages and monitor significant changes in phage taxonomy [34]. In this study, each selected phage could be classified as a myovirus, based on the presence of an icosahedral head and a long contractile tail. Biosafety assessment of phages for therapeutic purposes, such as analysis of their genomes, is also required. Phages carrying antibiotic resistance genes, virulence genes, and lysogenic modules should be avoided because they can transfer these factors to their hosts, which might exacerbate the adverse effects of pathogenic bacteria.

## Conclusion

This paper presented the properties of four newly isolated *E. coli* phages able to infect various *E. coli* strains. For the application of phages, the appropriate phage titer should be considered to effectively eliminate bacterial cells and not promote the development of phage-resistant bacteria. The results obtained in this study revealed the potential of phages as an alternative treatment for swine enteric colibacillosis. However, care should be taken when selecting phages for treatment to effectively control specific bacterial infections.

## Authors’ Contributions

RN, YW, and SS: Designed and supervised the study. TS, NI, and PS: Materials and methods preparation. TS and SS: Data collection and analysis. TS, NI, and SS: Drafted the manuscript. All authors conducted and commented on the manuscript. All authors have read and approved the final manuscript.
